# The burden of unlawful use of opioid and associated epidemiological characteristics in Africa: A scoping review

**DOI:** 10.1371/journal.pone.0317036

**Published:** 2025-03-07

**Authors:** Hope Onohuean, Frasia Oosthuizen

**Affiliations:** 1 Biopharmaceutics Unit, Department of Pharmacology and Toxicology, Kampala International University Western Campus, Ishaka-Bushenyi, Uganda; 2 Discipline of Pharmaceutical Sciences, School of Health Sciences, Westville Campus, University of KwaZulu-Natal, Durban, South Africa; Kore University of Enna: Universita degli Studi di Enna 'Kore', ITALY

## Abstract

**Introduction:**

There is an ongoing global upsurge of opioid misuse, fatal overdose and other related disorders, significantly affecting the African continent, due to resource-limited settings and poor epidemiological surveillance systems. This scoping review maps scientific evidence on epidemiological data on unlawful opioid use to identify knowledge gaps and policy shortcomings.

**Method:**

The databases (PubMed, Scopus, Web of Sciences) and references were searched guided by Population, Concept, and Context (PCC) and PRISMA-ScR. The extracted characteristics examined were author/year, African country, epidemiological distribution, age group (year), gender, study design and setting, common opioid/s abused, sources of drugs, reasons for misuse, summary outcomes and future engagement.

**Results:**

A population of 55132 participated in the included studies of 68 articles, with the largest sample size of 17260 (31.31%) in a study done in South Africa, 11281(20.46%) in a study from Egypt and 4068 (7.38%) in a study from Ethiopia. The gender of the participants was indicated in 65(95.59%) papers. The mean and median age reported in 57(83.82%) papers were 15.9-38, and 22-31years. The majority of study-designs were cross-sectional, 44(64.71%), and the most used opioids were heroin, 14articles (20.59%), tramadol, 8articles (11.76%), and tramadol & heroin, 6 articles (8.82%) articles. Study-settings included urban community 15(22.06%), hospital 15(22.06%), university students 11(16.18%), and secondary school learners 6(8.82%). The highest epidemiological distributions were recorded in the South African study, 19615(35.60%), Egyptian study, 14627(26.54%), and Nigerian study 5895(10.70%). Nine (13.24%) papers reported major opioid sources as black market, friends, and drug dealers. To relieve stress, physical pain and premature ejaculation, improve mood and sleep-related problems and help to continue work, were the major reasons for taking these drugs as reported in twenty articles (29.41%).

**Conclusion:**

The findings of this scoping review show significant knowledge gaps on opioid usage in the African continent. The epidemiological distribution of unlawful use of opioids among young adults, drivers, and manual labourers in both genders is evident in the findings. The reason for use necessity scrutinises the role of social interaction, friends and family influence on illicit opiate use. Therefore, there is a need for regular epidemiological surveillance and investigations into multilevel, value-based, comprehensive, and strategic long-term intervention plans to curb the opioid problem in the region.

## Introduction

Opiate use disorders and overdoses are an emerging global health concern. Both prescriptions and non-clinical indications contribute to the escalating global opioid use disorder problem (OUD). The opioid crisis has metamorphosed through the Use of: methadone in 1999, heroin in 2010, and the current wave of a combination of heroin, counterfeit pills, and cocaine [1–8]. An estimated 62 million people globally used opioids in 2019, and 36.3 million were impacted by its associated problems [9]. In the US estimated use has increased from 70029 in 2020 to 80816 in 2021 [10], and in Canada, 7560 opioid-related fatalities occurred in 2021 [11]. In Italy opioid addiction affects more than five people per 1000 [12], while a regional study in Germany conducted amongst 57 million adults, found opioid prescription prevalence of 38.7 or 12.8/1000 persons of low- and high-potency opioids in 2020 [13]. However, little is known about the epidemiological characteristics in Sub-Saharan Africa.

There are reports of opioid abuse, although not specifically on opioid fatal overdose or its related disorders, in some African countries, including Egypt, Nigeria, Kenya, Tanzania, and South Africa [14–24]. Some of these studies report the increasing use of tramadol and heroin among university and secondary school students, factories and site workers, long-distance drivers, sex workers, as well as unemployed youth [14–16,23,24]. However, in many other African countries, there is scanty or no information regarding the ongoing opioid crisis.

The findings on the reason for illicit opioid use includes; pleasure-seeking, craving, habits, impulsivity, improving energy [25], relieving stress [26], peer pressure from friends [27], engendering “morale” and “courage” to engage in sex work and “fight” potentially abusive clients [28]. Some of the reported sources are the black market [29], friends and drug dealers [30], roadways, bus terminals or intercity stands, low-income residential areas, abandoned or unfinished buildings, and fishing camps along the Indian Ocean [31].

Global opioid trafficking channels exist from Afganistan, through the india ocean and East Africa to the west [19,32,33]. This impacts heroin use among the population living in the coastal region of Comoros, Tanzania, Kenya, northern Mozambique, Madagascar, Mauritius and Seychelles [34–36]. Unlawful use of opioids could aggravate the already sporadic spread of infectious diseases like malaria, cholera, and HIV [37–41]. In 2018, the UNODC [42,43] predicted with insufficient evidence that another opioid crisis was developing in Africa. Inadequate vital record-keeping and surveillance systems make it challenging to comprehend the incidence burden and effects of opioid overdose in Africa [44].

### The rationale for this scoping review

Globally, opioid overdose and deaths are increasing, but little is known about these issues in resource-limited regions of Africa. In Africa, the burden of unlawful use of opioids, overdose deaths, and other disorders remains unquantified, and knowledge and access to possible interventions are generally limited. Again, many poor resource settings in Africa lack reliable vital records, and limited surveillance and epidemiological data have limited our understanding of the prevalence and consequences of the opioid crisis in the region [45]. Opioid issues may be hitting hard on the African continent in combination with the accelerated spread of infectious diseases like HIV [46,47], and the lack of resources available to solve these concerns may impede future advancement towards challenging objectives of the Joint United Nations Programme on HIV/AIDS [48–50]. A Scoping review on the burden of unlawful use of opioids and epidemiological characteristics in Africa will highlight the contributing drivers, public health risk factors and reason for use for effectiveness in control/management and intervention of the brewing opioid crisis in the region. The study aims to synthesise scientific evidence on epidemiological data on unlawful use of opioid/ abuse, the contributing drivers, the reason for use, sources and the impact on public health in the region.

## Materials and methods

The study was conducted to answer the questions on evidence available from the existing literature on epidemiological data on unlawful use of opioid/ abuse, the contributing drivers, the reason for use, and prevention and management on the ongoing opioid-overdose crisis epidemic. The scoping review was guided by the Population, Concept, and Context (PCC) approach developed by Joanna Briggs Institute (JBI) [51,52], details in step outline in the protocol (Onohuean and Oosthuizen 2024).

The ***population*** of interest (all African countries or within the African continent), the ***concept*** covers (epidemiological data, interventions and outcomes). Details include the epidemiological characteristics, prevalence, incidences of unlawful use of opioid/ abuse, the contributing drivers, the reason for use, sources and the impact on public health in the region. Also, reported informations on evidence-based drugs medication of opioid use disorder (MOUD), or Medication-Assisted Treatment (MAT) that is the FDA-approved medication used in conjunction with a psychosocial intervention such as (methadone, buprenorphine and naltrexone) [53,54], evidence-based interventions to prevent the spread of blood-borne pathogens associated with opioids overdoses, such as needle and syringe programs (NSPs), psychoeducation program, Recovery Solutions for Opioid Patients (RSOP) [55], campaigns or advocacy or training, opioids surveillance, prescription drug monitoring programs, findings/conclusions and any additional interventions mentioned by the authors of the studies included were reviewed and meta-synthesised. The ***context*** refers to studies that were undertaken in Africa. ***Types of evidence sources*** were sources of evidence that cover studies irrespective of study design, such as quantitative studies (e.g., experimental, quasi-experimental, prospective and retrospective cohort, case-control, cross-sectional, community or population-based), observational studies (e.g., case series, individual case reports, descriptive cross-sectional studies), qualitative studies, and mixed-methods studies [52,56,57].

### Search strategy

The keywords relevant to the overarching questions’ characteristics were developed to retrieve data on epidemiology, prevalence, opioids and types as details in the supplementary file (Text A1). The keyword was searched in title specific on the three databases; PubMed, WOS, Scopus and other references.

### Study selection

The Preferred Reporting Items for Systematic Reviews and Meta-Analyses extension for Scoping Reviews (PRISMA-ScR) were followed for the study selection process [58]. All identified research articles written in English from 1990 till 2022 were retrieved on 25 June 2022 at 2:15 PM and updated on 17 December 2022 at 12:05 AM. The data set was merged and normalised in ScientoPy and fBasics R-packages, and duplicates were removed and compiled to save in CSV or Excel file [59–61].

### Inclusion and exclusion criteria

The following qualifying requirements were applied in order to include studies: research that was done in the Africa continent, reported on any opioids such as natural (morphine, codeine, and thebaine (paramorphine); semi-synthetic (hydromorphone, hydrocodone, oxycodone, and heroin); fully synthetic (fentanyl, pethidine, levorphanol, methadone, tramadol, and dextropropoxyphene), or reported opioids with other substance or in combination were eligible for inclusion. Also, studies that report mainly on the outcomes of unlawful opioid use such as incident, prevalence, mortality rates, illicit uses and opioids identified by Diagnostic and Statistical Manual of Mental Disorders (DSM-5) codes or urinary analysis, log book, law enforcement or police record, toxicological investigation, autopsy, postmortem, or determined by Substance Abuse and Mental Health Services (SAMHSA). However, research meeting abstracts, proceedings papers, editorial materials, notes, letters, review or mata-analysis review articles, early access, book chapters, news items, reprints, conference paper, short survey, opioids used in cancer treatment, opioid study based on animal experiments, studies that has no total population or cases, other substance abuse without any opioid uses, opioid associated with Covid 19 or HIV or cancer or any terminal illiness, articles on machine learning, other languages not English, e.g., French were not qualified and were excluded. While, there were no limitations to study design or method or sample size or age or sex or type of opioids and along with other drugs.

### Data extraction

The two authors (HO and FO) separately examine the titles and abstracts of the retrieved dataset for possibly suitable papers. Studies that where not conducted in Africa, or literature reviews, systematic reviews, conference abstracts, and opinion publications, were omitted. The selected studies’ full texts were carefully examined, and the findings corresponding to the scoping review objective(s) and the information on authors, year of publication of the article, the country where the study was conducted, study design and setting, epidemiological characteristics, prevalence, age group (year), sex, common opioid abuse, sources of drugs, reasons, type of intervention/ summary outcomes/conclusions extracted in the data extraction form. At the same time, issues over eligibility were resolved through debate and consensus among authors. The primary research investigators were contacted when necessary to get any missing data/information from their studies.

## Results

### Literature search summary

**Fig 1:**
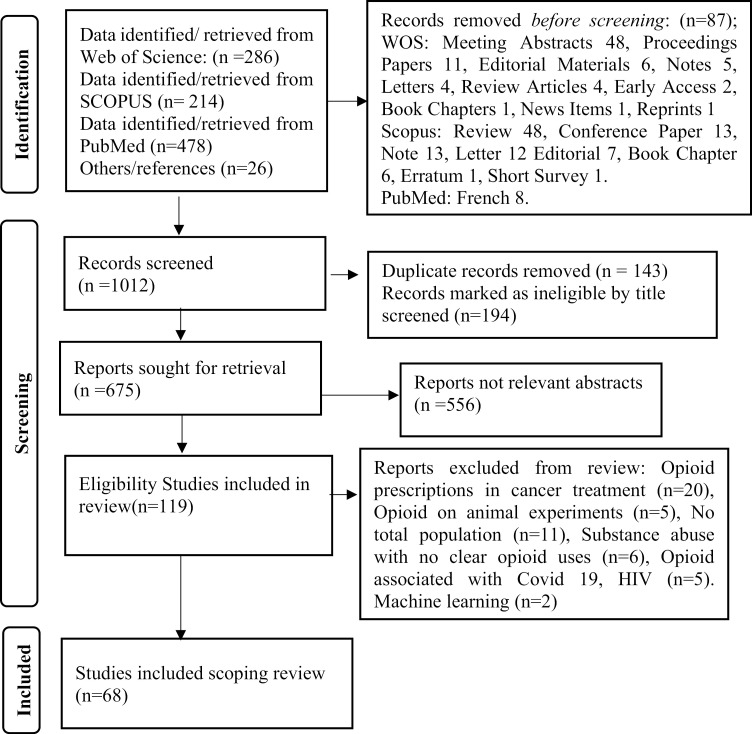
PRISMA study selection flowchart in the scoping review.

Sixty-eight (n = 68) eligible articles were included in the meta-synthesis in this study, while 49 papers were excluded at full-text review, including papers on opioid prescriptions in cancer treatment (n = 20), opioid on animal experiments (n = 5), articles with no report of the population (n = 11), substance abuse with no apparent opioid uses (n = 6), opioid associated with Covid 19 and HIV (n = 5), and publication using machine learning (n = 2) as depicted in Fig 1.

### Included studies characteristics

Between 1990 and 2022, the number of published articles in the African region has increased, and most 50 (73.53%) of the papers presented in the scoping review were published within the last 12 years. A total population sample of 55132 participated in these studies, with the largest sample size of 17260 (31.31%) reported in South Africa [62], 11281 (20.46%) in Egypt [63] and 4068 (7.38%) Ethiopia [64]. The gender of the participants was indicated in 65 (95.59%) papers, and breakdowns were 5 (7.35%) male and 7 (10.29%) female, and 53 (77.94%) both genders. The age bracket reported in 57 (83.82%) papers was mean, median age ranged between 15.9 and 38 years, and median age 22 and 31, as detailed in Table 1.

**Table 1:** Overall characteristics of the studies meta-synthesised in the scoping review.

Author/year	Year	Countries	Design	Sample	Setting	Age group (year)	Sex	Common Unlawful use opioids and other drugs	Sources of drugs	Reasons
[65]	2018	Cameroon	Cross-sectional survey	852	Medical and nursing schools	Mean age 21.78	Both	Tramadol and marijuana	N/a	N/a
[26]	2022	Cameroon	Cross-sectional survey	650	University students	15-64	Both	Tobacco, alcohol, tramadol and cannabis	Peers	Substance use was to relieve stress
[66]	2001	Egypt	Hospital population	200	Hospital	Adults	Both	Heroin, benzodiazepines	N/a	N/a
[67]	2021	Egypt	Identification test and urine screen	122	Hospital	Adolescents	Male	Tramadol and heroin	N/a	N/a
[25]	2019	Egypt	Cross-sectional observational study	100	Hospital	Mean age 28.5	Male	Tramadol and heroin	N/a	Pleasure seeking, craving, habits, impulsivity, improving energy, staying awake, and Deepen insights.
[68]	2013	Egypt	Cross-sectional survey	451	Hospital	N/a	Both	Tramadol and cannabis	N/a	N/a
[30]	2020	Egypt	Interview questionnaire	900	Drivers, construction and textile industries workers	Mean age 30.93	Both	Tramadol	Friends and drug dealers	Improve mood, relief of pain and help to continue work
[63]	2022	Egypt	Retrospective study	11281	Treatment centre	20-40	Both	Tramadol, cannabis	N/a	N/a
[69]	2015	Egypt	Identification test and urine screen	204	Secondary school	13-18	Both	Tramadol	N/a	N/a
[70]	2016	Egypt	Interview questionnaire	100	Hospital	Mean age 30.3	Both	Tramadol	N/a	N/a
[29]	2018	Egypt	Cross-sectional study	1173	University students	Mean age 17.6	Both	Tramadol	Black market	Physical, like pain and premature ejaculation or psychological, like mood or sleep-related problems
[71]	2022	Egypt	Identification test and urine screen	3	Laboratory	Mean age 23.1	N/a	Cannabinoid fub-amb (methyl(2s)-2-{[1-[(4-fluorophenyl) methyl]indazole-3-carbonyl]amino}-3 methylbutanoate). Voodoo containing mixture of tramadol, methadone, morphine derivatives,	Voodoo	N/a
[27]	2015	Egypt	Focus groups	40	Public	12-14 & 15-18	Both	Tobacco, alcohol, tramadol	N/a	Peer pressure from friends stood out as the most common reason to start and continue using substances,
[72]	2015	Egypt	Identification test and urine screen	53	Hospital	Mean age 23.1	Both	Tramadol	Black market	N/a
[73]	2017	Ethiopia	Exploratory mixed methods study	102	Community	N/a	Both	Khart, Heroin	N/a	N/a
[74]	2018	Ethiopia	Cross-sectional survey	237	N/a	>15	Both	Heroin, marijuana or ganja and/or khat	N/a	N/a
[64]	2020	Ethiopia	Identification test and urine screen	4068	N/a	>18	Both	Heroin, alcohol, khat, and cannabis	N/a	N/a
[75]	2019	Ethiopia	Cross-sectional survey	586	University students	20–24	Both	Lysergic diethylamide (LSD), cocaine, crack, heroin,	N/a	‘Peer influence’ and ‘to get energised for study.’
[76]	1999	Ghana	Interview questionnaire	116	Psychiatric hospital	18-32	Both	Heroin, marijuana, cocaine	N/a	N/a
[77]	1999	Ghana	Exploratory study	117	Community	19-50	Both	Heroin and cocaine	N/a	N/a
[16]	2021	Ghana	A cross-sectional study, mixed-methods	458	Commercial drivers and assistants	Mean age 25.1	Both	Tramadol	N/a	Pain relief and sexual enhancement.
[78]	2020	Ghana	Cross-sectional survey	171	Hospital	>18	Both	Alcohol, marijuana, oxycodone, cocaine, diazepine, methamphetamines	N/a	N/a
[79]	2015	Kenya	Cross-sectional survey	1785	Community	N/a	Both	Heroin	N/a	N/a
[80]	2015	Kenya	Identification test and urine screen	186	Kenyan coast	Mean age 33	Both	Heroin	N/a	N/a
[81]	1999	Kenya	Cross-sectional survey	558	University students	Age range 16-50	Both	Tobacco, alcohol, cannabis, heroin	N/a	N/a
[82]	2021	Kenya	Cross-sectional survey	646	Community	11- 53	Both	Heroin, cocaine	N/a	N/a
[83]	2015	Kenya	Cross-sectional survey	151	Community	Mean age 28.8	Both	Alcohol, marijuana, cocaine, heroin, brown sugar,	N/a	N/a
[28]	2019	Kenya	Cross-sectional survey	45	Sex worker	Median age 28	Female	Heroin	N/a	Engender “morale” and “courage” to engage in sex work and “fight” potentially abusive clients
[84]	2016	Kenya	Conducted interview-administered surveys	151	Community	Mean age 28.8	Both	Heroin and cocaine	N/a	N/a
[85]	2005	Kenya	Ethnographic	26	N/a	N/a	Female	Heroin	N/a	N/a
[86]	2006	Kenya	Ethnographic	496	N/a	N/a	Both	Heroin	N/a	N/a
[87]	2008	Nigeria	Interview questionnaire	400	Secondary school students	Mean age 17.1	Both	Kolanuts, tobacco/cigarettes, alcohol, Indian hemp, cocaine, tramadol	N/a	
[88]	2005	Nigeria	Interview questionnaire	1200	University students	10-19	Both	Kolanut and coffee, alcohol, sniffing agents, amphetamine and ephedrine, cigarette, heroin, cocaine and cannabis	N/a	N/a
[89]	2001	Nigeria	Interview questionnaire	750	Secondary school	N/a	Both	Alcohol, cigarettes, cannabis, strong and mild stimulants, hypnosedatives, antibiotics, cocaine, heroin, organic solvents and hallucinogens	N/a	N/a
[90]	1991	Nigeria	Cross-sectional survey	62	Neuropsychiatric hospital	N/a	Both	Marijuana, alcohol, heroin and cocaine	N/a	Unemployment, poor educational status
[91]	2020	Nigeria	Cross-sectional survey	784	University students	Median age 22	Both	Codeine, marijuana, and tramadol	N/a	Cultural expectations
[92]	2021	Nigeria	Cross-sectional survey	93	Local government	19-53.9	Both	Alcohol, cigarettes, marijuana and codeine	N/a	Peer pressure and low level of national regulations
[93]	1995	Nigeria	Cross-sectional survey	55	Rehabilitation camp	Mean age 30.6	Both	Heroin and cocaine	N/a	N/a
[94]	2018	Nigeria	Cross-sectional survey	249	Secondary school	9-28	Both	Alcohol, cigarette smoking heroin, cocaine and marijuana	N/a	Enhance their academic performance
[95]	2019	Nigeria	Cross sectional study	224	Military officers	N/a	Both	Heroin	N/a	N/a
[96]	2012	Nigeria	Cross-sectional survey	557	Hospital	15- 48	Female	Kolanuts, chlorpheniramin, alcohol, diazepam and promethazine, cigarettes/tobacco, phenobarbitone, cocaine, codeine, and marijuana.	N/a	Control nausea and vomiting of early pregnancy.
[97]	2022	Nigeria	Qualitative survey	15	Hospital	N/a	Male	Flunitrazepam (street name blueboy, sweetnol), trihexyphenidyl, codeine and tramadol	N/a	N/a
[98]	2010	Nigeria	Cross-sectional survey	402	Secondary school students	Mean age 15.9	Both	Caffeine, heroin and cocaine	N/a	Relief from stress, self-medication to treat illness and to stay awake at night to study
[99]	2021	Nigeria	Ethnographic research design	50	Community	<18 - 45	Both	Tramadol and cannabis, morphine, heroine, methadone, buprenorphine, codeine, oxycodone and hydrocodone	N/a	Enhance their farming livelihood practices, to withstand stress during farming
[100]	2017	Nigeria	Cross-sectional survey	233	Hospital	Adults	Both	Codeine, cannabis, cocaine and alcohol	N/a	N/a
[101]	2021	Nigeria	Cross-sectional survey	301	University students	Mean age 22.6	Male	Tramadol	N/a	Relieving tiredness and prolong sex. Cheap prices and ease of access
[102]	2019	Nigeria	Cross-sectional survey	520	University students	Mean age 20.7	Both	Codeine	Pharmacy, internet and patent medicine vendors	N/a
[62]	2015	South Africa	Cross-sectional survey	17260	Community	Mean age 25.3	Both	Codeine	N/a	N/a
[31]	2008	South Africa	Cross-sectional survey	52	Community	15-41	Both	Heroin	Hotspots; roadways, concentrating near bus terminals or intercity stands, low-income residential areas, abandoned or unfinished buildings, and fishing camps along the Indian ocean.	Ecstasy stimulant enhance libido (female sex worker), injection enhance sexual experience, snorting prolongs sexual stamina, anal administration build confidence (male).
[103]	2009	South Africa	Cross-sectional survey	77	Nation wide	18-55	Both	Cocaine, ecstasy, heroin and methaqualone	N/a	N/a
[104]	2019	South Africa	Cross-sectional study	401	Community	Median age 38	Both	Heroin	N/a	N/a
[105]	2020	South Africa	Retrospective study	1513	University students	Median age 30	Both	Heroin, methadone	N/a	N/a
[106]	2019	South Africa	Cross-sectional survey	78	University students	>18	Female	Cannabis, lysergic acid diethylamide [lsd], magic mushroom, cocaine, crack, ecstasy, methamphetamine and heroin, methylphenidate	N/a	Adjusting to a new social environment or adapting to the academic load and responsibilities, peer pressure, social activities and external factors
[44]	2022	South Africa	Cross-sectional survey	66	Community	Median age 31	Male	Heroin	N/a	N/a
[107]	2017	South Africa	Case presentations	2	N/a	N/a	Male	Heroin	N/a	N/a
[108]	2017	South Africa	Interview questionnaire	25	Community	Adult	Both	Codeine	N/a	N/a
[109]	2014	South Africa	Cross-sectional survey	141	Hospital	Mean age 26.5	Both	Heroin	N/a	N/a
[110]	2020	Sudan	Cross-sectional survey	317	Medical school	Mean age 21.5	Both	Cannabis, cocaine, and heroin	N/a	N/a
[111]	2016	Sudan	Cross-sectional survey	500	University students	15 - 64	Both	Tobacco, cannabis, alcohol, amphetamines, tranquilisers, inhalants, opiates, cocaine, and heroin	Peers	Curiosity reason for initiation of substance use, pleasure, relief of psychological stress, and relief of fatigue
[112]	2011	Tanzania	Cross-sectional survey	184	Hospital	15 - 80	Both	Alcohol, tobacco cannabis, heroin and cocaine	N/a	N/a
[113]	2010	Tanzania	Cross-sectional survey	169	Hard-to-reach populations	N/a	Female	Heroin, marijuana,	N/a	N/a
[114]	2018	Tanzania	Cross-sectional survey	436	Regional	18-54	Both	Cocaine and heroin	N/a	N/a
[33]	2018	Tanzania	Cross-sectional survey	200	Urban community	18-40	Female	Heroin	N/a	N/a
[115]	2021	Tanzania	Cross-sectional survey	200	Urban community	18 above	Women	Heroin	N/a	N/a
[21]	2015	Tanzania	Interview	480	Urban community	Mean age 28.8	Both	Heroin	N/a	Peer pressure, for fun, family problems and influence from family
[22]	2018	Tanzania	In-depth interviews	436	Regional	Mean age 25.3	Both	Heroin	N/a	N/a
[116]	2016	Tanzania	Cross-sectional survey	288	Hospital	18 above	Both	Heroin, cocaine, methadone	N/a	N/a
[117]	2021	West Africa	Cross-sectional survey	384	Secondary school students	10-24	Both	Tramadol	Street-level markets	N/a

N/a: Not reported.

### Study design

The majority of the studies had a cross-sectional observational study and cross-sectional study design 44 (64.71%), were as informed interview questionnaire 9 (13.24%), experimental conformation identification test and urine screen 6 (8.82%), ethnographic research design 2 (2.94%), retrospective study 2 (2.94%), mixed exploratory methods study 2 (2.94%), secondary data collection by qualitative survey 1 (1.47), focus groups 1 (1.47%), case presentations 1 (1.47%) Table 1.

### Study setting

Sixty-four articles of the reported studies indicate the study settings. Majority of the studies settings include; community and urban community 15 (22.06%), hospital (neuropsychiatric hospital and Psychiatric hospital) 15 (22.06%), university students 11 (16.18%), secondary school students 6 (8.82%), commercial drivers and assistants, construction and textile industries workers 2 (2.94%), medical school students 2 (2.94%), regional survey 2 (2.94%), rehabilitation camp and treatment centre 2 (2.94%), laboratory 1 (1.47%), local government 1 (1.47%), military officers 1 (1.47%) nation-wide survey 1 (1.47%) general public 1 (1.47%) sex worker 1 (1.47%), hard-to-reach populations 1 (1.47%), Kenyan coast 1 (1.47%) Table 1.

### Epidemiology of unlawful opioid use in the African region

**Fig 2:**
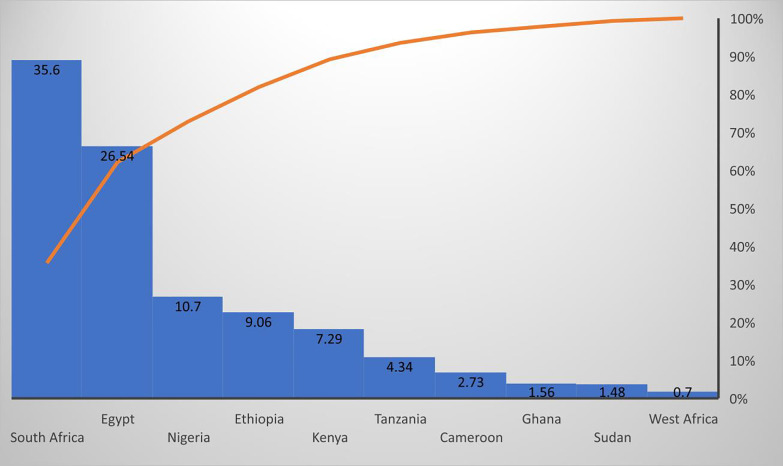
Distribution of opioid uses among the African countries of the meta-synthesised studies in the scoping review.

In many African countries, misuse of opioids and other drugs continues to grow with little mainstream research notice, while some regions have no reported data. The epidemiological distributions of the majority of the identified articles were reported in nine Africa countries and West Africa (mixed countries), including South Africa 19615 (35.60%), Egypt 14627 (26.54%), Nigeria 5895 (10.70%), Ethiopia 4993 (9.06%), Kenya 4018 (7.29%), Tanzania 2393 (4.34%), Cameroon 1502 (2.735), Ghana 862 (1.56%), Sudan 817 (1.48%), West Africa 384 (0.70%) Fig 2.

### Commonly unlawful used opioids in the African countries

**Fig 3:**
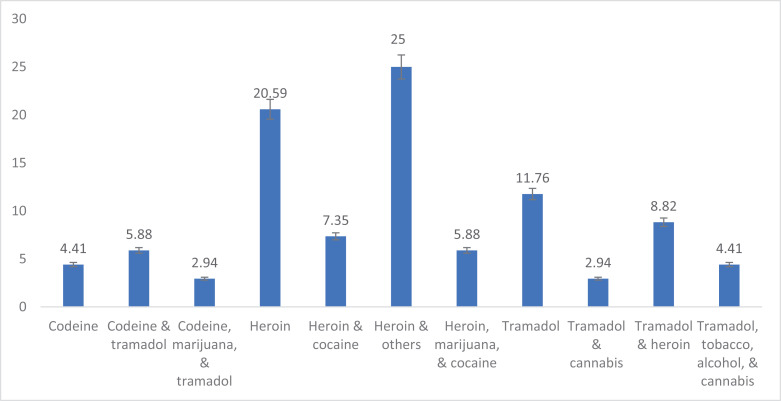
Percentage distribution of the commonly unlawful used opioids and other drugs among the meta-synthesised studies in the scoping review.

The most commonly abused opioids reported in the included 68 studies are as follows: 14 (20.59%) papers reported misused of heroin only, heroin & others (alcohol, tobacco, cannabis, cigarettes, marijuana, khat, caffeine, magic mushroom, methylphenidate) were reported in 17 (25%) papers, heroin & cocaine was presented in 5 (7.35%), only tramadol 8 (11.76%), and tramadol & heroin documented in 6 (8.82%) articles details in Fig 3.

### Sources of opioids drugs among the African countries

Most studies do not indicate the sources of opioid use in the reported studies of African countries. However, only nine (13.24%) papers reported sources such as the black market, friends and drug dealers being the major source of tramadol [29,30,72]. Heroin hotspots include roadways, concentrating near bus terminals or intercity stands, low-income residential areas, abandoned or unfinished buildings, and fishing camps along the Indian ocean [31]. According to the report of [26,111], tramadol, tobacco, cannabis, alcohol, amphetamines, tranquillisers, inhalants, opiates, cocaine, and heroin were sourced by peers in most regions. Codeine is sold by retail pharmacies, internet and patent medicine vendors [102]. Other sources for tramadol were street-level markets [117]. Cannabinoid, fub-amb (methyl(2s)-2-{[1-[(4-fluorophenyl), methyl] indazole-3-carbonyl]amino}-3 methyl butanoate) and mixed opiates where gotten by Voodoo [71].

### Reasons for opioids use among the African countries

Twenty (29.41%) papers assessed the reason for unlawful use of opioids in the African region. The study by [26] in Cameroon, indicate substance use was to relieve stress. The major reason for opioid use covered in Egyptian studies included; physical, e.g., pain and premature ejaculation or psychological, e.g., mood or sleep-related problems [29]. The study by [30] highlighted improved mood, relief of pain and help to continue work. In the study by [27], peer pressure from friends stood out as the most common reason to start and continue using substances. Also, [25] reported pleasure-seeking, craving, habits, impulsivity, improved energy, staying awake, and deepening insights as the proper factors for opioid use. Similar reasons of peer influence and getting energised, were reported by [75] in Ethiopia. The study in Ghana reported pain relief and sexual enhancement as the main purpose for use [16]. However, the report of [28] in Kenya highlighted “engender “morale” and “courage” to engage in sex work and “fight” potentially abusive clients”. The report from studies in Nigeria’s literatures were not so different from other countries in the region. Among them were to control nausea and vomiting during early pregnancy [73], cultural expectations [91], to enhance their academic performance [94], to enhance their farming livelihood practices and withstand stress during farming [99]. Also, peer pressure and low level of national regulations [92], while relief from stress, self-medication to treat illness and staying awake at night to study was reported by [98]. Again, relieving tiredness and prolonged sex, cheap prices and ease of access were documented by [101], while [90] reported unemployment and poor educational status as the reason for use. In South Africa, the study of [106] documented adjusting to a new social environment or adapting to the academic load and responsibilities, peer pressure, social activities and external factors. Ecstasy stimulants enhance libido (female sex worker), injection enhances the sexual experience, snorting prolongs sexual stamina, and anal administration builds confidence (male) were reported by [31]. The study by [111] in Sudan reported curiosity as the reason for the initiation of substance use, pleasure, relief of psychological stress, and relief of fatigue. In Tanzania, similar reasons, including peer pressure, fun, family problems and influence from family, were motives for commonly used [21].

### Summary outcomes and future engagement on the burden of unlawful use of Opioid and epidemiological characteristics in Africa

The summary outcomes and future engagement is highlighted in Table 2. Future studies may focus on the elements that influence incentives to change in regard to illicit opioid drug use and are likely to focus on views of the impact on social relationships, the social context of the situation, and the behaviours of friends and family that influence the patients to think about changing. The findings of this scoping review have presented the suggestion of investigators that multilevel, value-based, comprehensive, and strategic long-term intervention plans are some requirements for curbing the opioid problem [26]. Population-based longitudinal studies are needed to investigate tramadol use and the possible role of tramadol as a gateway drug [69]. Expanding coverage of current laws and occupational safety and health standards to cover workers in the informal sector, especially in developing countries, was recommended by Abbas et al., 2013 [68]. Deyessa et al., 2020 [64] suggested the implementation of the essential harm reduction strategies given by the world health organisation. Community awareness and training on needle syringe programs (NSP), and assisted therapy (MAT) were proposed by Oguya et al., 2021 [82]. Lingering on access to naloxone to lay people and community and peer-based opioid overdose prevention training in Tanzania was recommended by Saleem et al., 2021 [33], which could also be spread to other Africa nations. Psychiatric comorbidities associated with heroin use disorders patients underscore the need for substance treatment services with the capacity to diagnose and manage these comorbidities [109].

**Table 2:** Summary findings for future engagement on the Burden of Unlawful Use of Opioid and Epidemiological Characteristics in Africa.

Author/year	Outcomes and future engagement
[67]	Attention-deficit/hyperactivity disorder (ADHD) is common among adolescents with tramadol misuse. There is an association between ADHD and the young age of onset of tramadol misuse and drug-related problems.
[26]	Multilevel, value-based, comprehensive, and strategic long-term intervention plans are required to curb this problem.
[65]	The use of recreational drugs by young adult may negatively impact their career performance and health.
[69]	Over one-third of tramadol users had drug-related problems, while population-based longitudinal studies investigating tramadol as a gateway drug.
[30]	Industrial workers reveal the reasons related to the work load and stressful events for unlawful tramadol use. Workplace drug testing may ensure community safety.
[68]	Expanding the coverage of drug policy to occupational safety and health standards to cover workers in the informal sector is recommended.
[27]	Focus on quantitative surveys to estimating the prevalence of specific behaviours related to substance use among youth may be a potential avenues for prevention.
[71]	Voodoo is a mixture of substances of abuse at varying concentrations.
[63]	Tramadol and cannabis causes acute intoxication, resulting from recreational use/misuse of prescription drugs.
[75]	There is high prevalence of alcohol, khat, cigarettes, and marijuana uses among undergraduate University students. Influenced by living alone during school age, being male, family and friends who used drug, appear to be independent predictors of drug use among undergraduate.
[64]	Lack of service in harm reduction in the city of Addis Ababa has made PWIDS vulnerable and at higher risk for HIV/Aids and hepatitis b and c. Need for implementing the essential harm reduction strategies.
[74]	Needle-sharing prevalence is high in Addis Ababa that requires the establishment of harm reduction programmes and prevention strategies for PWID in Addis Ababa.
[73]	A rigorous methodology that could test the gateway hypothesis of problematic psychoactive substance use could be warranted.
[78]	Alcohol and substance use (ASU) may be a preventable cause of adult injuries.
[77]	Noted a changed patterns of international drug trafficking in most sub-Saharan countries. Heroin, cocaine, and other psychotropic substances have exceeded marijuana in popularity. The sense of hopelessness-which is often attributed to the drug culture, and as a symptom of addiction-but many have to committed crimes to support it.
[80]	Coinfection screening, treatment-as-prevention for HIV and HCV and harm reduction should be scaled up to alleviate the infection burden associated with unlawful opioid uses.
[81]	The findings suggest an urgent need to gather more data, which can be used to guide the formulation of health promotion and prevention programmes.
[82]	The results show that people who inject drugs (PWIDS) are at particularly high risk of infection in Kenya, and intervention is urgently needed.
[79]	Implementing public health scale combination HIV prevention can potentially limit the epidemic in this vulnerable and bridging population significantly suggested in Nairobi.
[86]	The assessment results highlight the need for various services, including needle exchange, counselling, and referral to residential treatment programs recommended in Kenya.
[87]	Need to strengthen the monitoring and preventive programmes to reduce their spread in schools.
[98]	It is advocated that there is a need to review existing health educational programmes.
[100]	The prevalence of ADHD in SUD individuals is high and may be associated with a more severe phenotypic expression of SUD.
[93]	To curtail the growing numbers of displaced young people in the country, the government should pay attention to the root causes of the multiple social ills in society.
[92]	It is recommended that more education, an increase in awareness of the national regulations and peer-modelling techniques should be strengthened within the community to correct the negative perception by these groups of people.
[91]	Engagment of family to sensitise young people on the harmful effects of drug use. Is vital that religious leaders speak against drug use in their various fellowships. There is a need to address recreational drug use on Nigerian campuses by educating students about its adverse impacts.
[99]	The enforcement of abuse law and establishment of rehabilitation centre for the correction and reformation of addicts in the state.
[88]	Using licit and “socially” acceptable drugs may pave the way for the abuse of illicit ones.
[90]	Ensuring proper rehabilitation and follow-up for opioid drug abusers.
[94]	Suggest an urgent need to intensify awareness against substance abuse among secondary school students in Nigeria.
[97]	Newer substances of abuse in their various combinations are abused by Nigerian youth. More studies are needed to elucidate further the chemical composition of these drugs/mixtures and their mechanism of action.
[101]	Intervention programs targeting reducing ACEs’ impact on TU should recognise the need to address sociosexuality inclinations to achieve better intervention outcomes among tramadol-using students.
[44]	Increased awareness of actions to undertake in response to an overdose (calling for medical assistance, using naloxone) and access to naloxone are required. Additional data are needed to better understand the nature of overdose in South Africa to inform policy and responses.
[106]	Additional student support, early identification and referral for management and/ or rehabilitation should be a priority at tertiary institutions responsible for training future healthcare professionals.
[103]	Interventions recognising the role of drug abuse in HIV transmission should be prioritised, and issues of access to services, stigma and power relations must be considered.
[118]	The study underscores the need for continued support for enhanced patient awareness of the risk of habit-forming use, related health consequences, and professional pharmacovigilance.
[107]	An awareness program regarding this grave condition is planned. Originality/value - the cardiovascular complications of patients inhaling heroin vapour has not been highlighted previously.
[111]	Strategies to control substance use should encompass the role of the university and parents in observing and providing education to improve awareness of substances and their consequences.
[110]	Students’ perception of psychoactive substance use seems favourable, but increasing awareness is still recommended.
[33]	Overdose surveillance efforts and further work to characterise overdose risks in this context to design relevant, targeted interventions to prevent opioid overdose in Sub-Saharan Africa.
[115]	In Tanzania there is a need for expanded access to naloxone to lay people and community and peer- based overdose prevention training.
[112]	Need for appropriate standard of operations to be adopted as a matter of standard clinical practice and policy in psychiatric hospitals.
[116]	Aspects of mental and physical health, risk behaviours, and quality of life among drug users are intertwined and complex. Comprehensive supportive services and the provision of methadone are needed to address the complex health needs of people who inject drugs.
[117]	Report the nonmedical use of tramadol affects young adults in Benin, having a considerable concern among secondary school students.
[109]	Patients with heroin use disorders present with high rates of psychiatric comorbidities, which underscores the need for substance treatment services with the capacity to diagnose and manage these comorbidities.

## Discussion

This scoping review is done at a time when there is a global upsurge of opioid misuse and other related disorders, but little mainstream research attention is paid to the problem on the African continent, especially in resource-limited settings. However, we are confident that mapping the breadth and depth of research on the unlawful use of opioid will trigger stakeholders and government response in the African region. Here, we examine a growing corpus of research on the unlawful use of opioids and exclude opioid prescriptions or medications. We did not attempt to evaluate the quality of the included research articles since the scoping review made no assertions or conclusions about the findings. However, the gaps in the synthesised scientific evidence covering over three decades could aid in directing researchers and policymakers in managing the opioid crisis in Africa.

### Epidemiology of unlawful use of opioid in the Africa population

We found 68 original research papers discussing different illicit opioid use in Africa between 1990 and 2022 (32 years). The majority of the studies were from West Africa (Nigeria), Central (Egypt), East Africa (Keyan and Tanzania), and Southern (South Africa) regions. It affects all ages. The rising illicit use of opioids and other drugs is poorly documented in Africa with zero statistics.. The paucity of epidemiologic information about unlawful use of opioid and overdose in the literature reviewed making it difficult to understand the opioid crisis in Africa.

We found few published articles on the prevalence of opoid usage, but these lack the detail of overdose crisis [2,119,120]. The methodological approach for opioid research in Africa is constrained and most of the assessment window’s timing, population and the tools employed varied. A study on Nation-wide, secondary school and university students, rural and urban communities, and hospitals, showed the evolving consequences of unlawful use of opioid and related disorders. Additionally, it was uncommon for most studies to distinguish between occasional opioid usage (e.g., tried many times in the past week) and single-use (e.g., tried once in a lifetime), and verified clinical diagnoses were not published at all. Most publications were cross-sectional investigations without effort at participant follow-up or extended beyond the cohort. Also, few studies went beyond straightforward descriptions, correlations or risk factors, and it was uncommon for any underlying theories or conceptual models to be articulated. Lastly, a good number of studies focuses on people living with HIV and students as particularly high-risk groups, while scant evidence regarding unlawful use of opioid in other vulnerable population such as hard labourers, unemployed, internally displaced (refuges camp), students with co-occurring mental health issues etc.

### Demographic and burden of unlawful use of opioid in the African region

In Africa, the burden of unlawful use of opioid is not limited to specific demographics such as sex, age, occupation or population, only with some levels of variations, despite a variety of demographic, psychological, and social characteristics that have been linked to drug use in various studies [32,121,122]. The Sub-Saharan African region has been estimated to experience about a 130% increase in the burden of mental health, substance use disorders, and disability [32]. This main, further weekend, the already over-stressed health system with poor full-time equivalent (FTE) staffing needs for the health industry was estimated to be far lower than the actual FTE staffing levels recommended [123]. Nevertheless, due to a lack of estimated data and paucity of epidemiologic or clinical evidence on opioid overdoses, fatalities and other related disorders in the literature reviewed, the public health burden may be underreported. However, our findings highlighted the African nation’s distribution: young adults have been the most vulnerable population, and heroin, tramadol, and in combinations with other opioids or combination with other classes of substances are frequently misused in the region. This indicates a public health concern with other risk factors such as terrorism, violence, and the spread of infectious diseases, which require an urgent surveillance system, treatment/counselling centre and research centre for strategic prevention implementation.

Specifically, abuse of tramadol and codeine is a widespread, disturbing occurrence in the Egyptian community [25,30,72]; Nigeria [87,101], Ghana [16] particularly among secondary and university students, unemployed and long-distance drivers. Due to their easy availability on the over-the-counter (OTC), black market, low cost, and effectiveness in treating premature ejaculation, it became the most often used drug among young people and adults. However, studies reported that most work labourers and long-distance drivers admitted that tramadol helped them keep up with daily tasks, have a positive attitude, be more friendly, and not feel exhausted to accomplish challenging tasks and prevent withdrawal symptoms. This notion indicated a need for public awareness and re-orientation of the adverse effects of tramadol misuse and an awareness of regulatory authorities. At the same time, severe pain, myalgia, sleeplessness, and autonomic signs such as diarrhoea, rhinorrhea, lacrimation, and nausea were the most distressing side effects of tramadol withdrawal [72]. Heroin is also common misuse in Ethiopia [64,74], South Africa [105,107], and Western Kenya [79,80,86], while injecting heroin is becoming a more significant public health issue in Tanzania [21,28] with high potential of spreading to the country’s neighbouring regions, including Uganda, and Rwanda. Heroin trafficking and consumption have gradually increased in Tanzania over the past ten years. These implied that the East Africa nations must start assessing the likelihood of heroin use in their regions. Due to the increasing use of African trade routes by global opioid trafficking channels, the use of opioids for non-clinical purposes has increased in the continent [32,121,124].

The most alarming age population is 12-30. People in their midlife or potential life have been substantially connected with increased opioid fatal overdoses [125]. Opioid drug misuses such as morphine, codeine, hydrocodone, fentanyl, methadone, dextropropoxyphene, heroin, and tramadol, have now become a common component of adolescent experimentation, socialisation, risk-taking, and reward-seeking, especially done among a specific young group of people [126]. Sex workers often use opioid drugs such as heroin and fentanyl to enhance sexual performance, as a coping mechanism, to gain the courage to approach clients and endure the harsh street environment [126–128] with severe implication for sexually transmitted infections (STIs).

Looking at the age variability in our findings, the years spent in high school and traditional universities, differs [129,130] and there is a positive correlation between adolescence, adulthood and the emergence of substance use disorders due to adverse childhood experiences (ACEs) [131]. Also, self-reliance from parents, new communal and intimate connections alongside peers, increased access to drugs and alcohol, and the need for independent learning all give to a distinctive social environment which is at a phase of development that is characterised by the highest levels of risk-taking and its associated behavioural, mental health problems [132–134]. This is one of the certain utterly lacking crucial potential investigation areas based on this scoping review.

### The discrepancy between adequate use and need for opioids in Africa

The problem of unlawful use of opioids highlighted in this study is undeniably concerning, particularly in light of its impact on patients who genuinely require opioids for medical reasons, such as those suffering from cancer, HIV, palliative care, or terminal illness etc. While the study sheds light on the issue of illicit use, it is crucial to also recognize the significant problem of undertreatment of pain in many parts of the world, particularly on the African continent [135,136]. Also, reports and the official data from the United Nations and the World Health Organization (WHO) reveal the stark reality of the undertreatment of pain in Africa [137–139], where there is a scarcity and limited availability of opioids for medical use. This shortage of essential medications has dire consequences, leading to thousands of individuals enduring unnecessary suffering and experiencing inhumane deaths due to the lack of access to adequate pain relief.

While addressing the opioid abuse crisis is crucial, it is equally important to address the systemic barriers that prevent access to essential pain management medications for those who desperately need them. This underscores the urgent need for comprehensive strategies and policy interventions to ensure equitable access to pain relief medications for all individuals, regardless of their geographical location or socioeconomic status.

### Summary outcomes, prevention, treatment and future engagement

Our finding further shows an increasing illicit opioid drug use, which could soon reach or escalate to alarming levels in Africa, if not unnoticed, due to poor surveillance systems. Tramadol and heroin are commonly misused among adolescents in the region with the impact of attention-deficit/hyperactivity disorder (ADHD). However, our scoping review found insufficient published research on opioid drug-related harm reduction or prevention in the region. Psychological services or behavioural therapies for young stars and school students suffering from mental health issues associated with opioid abuse are completely lacking. This may be due to poor data from mainstream research for public awareness of the opioid crisis in the region or a result of the sluggish stakeholder response to unlawful use of opioid and its potential dangers, as well as the negative effects of stigma and legality on attempts to get help. Also, there is no report on clinical trials tailored towards delivering interventions to adolescence or adulthood-specific opioid issues in the region.

This review suggests more investigation is needed to comprehend better the mechanisms underlying adolescent and adulthood drug use and associated mental health issues

- Establishing a rehabilitation centre to correct and reform addicts or specific behaviours related to unlawful use of opioid at the early-stage diagnosis.
- surveillance initiatives and characterisation of overdose is recommended.
- Furthermore, multilevel, comprehensive, value-based, and strategic long-term intervention plans are needed.
- introduction of community peer-modeling strategies or techniques.
• the engagement of satkeholders in sensitise young people, about illicit opioid use and its adverse impacts.
- Research engaging relevant demographics in the design of interventions for opioid uses and other drugs of abuse may boost acceptability, adherence and implementation.
- brief in-person and motivational intervention delivery may be promising.
- Continuous tracking young star opioid drug and alcohol use is essential if effective evidence-based policy and intervention initiatives are to be developed.
- studies could be focused on prevalence and risk assessment of opioids abuse in the Africa continent as recommendation.
- evaluations of novel theoretically based prevention and treatment strategies that consider recognised risk factors for opioid use and disorders.

### Research limitations

Among the limitations of this scoping review is the restriction of articles written in English as mandated by inclusion criteria, while documents written in French were excluded. Additionally, no quantitative data analysis was performed in this study that could have questioned or supported our findings.

## Conclusion

Our scoping review has shown significant gaps in the body of research on opioid usage in the Africa continent. The findings are evident on the epidemiological distribution of the unlawful use of opioids among young adult, drivers and manual labor in both genders. Evidence-based healthcare assessment and regulations of the illicit sources or contributing drivers to inform prevention and management strategy. The reasons for use underscores the need to examining the social interactions impact, the actions of friends and family that influence unlawful opioid uses.

## Supporting Information

S1 File Preferred Reporting Items for Systematic reviews and Meta-Analyses extension for Scoping Reviews (PRISMA-ScR) Checklist. (DOCX)
